# Understanding and Implementing Diagnostic Stewardship: A Guide for Resident Physicians in the Era of Antimicrobial Resistance

**DOI:** 10.3390/microorganisms11092214

**Published:** 2023-08-31

**Authors:** Georgios Schinas, George Dimopoulos, Karolina Akinosoglou

**Affiliations:** 1 School of Medicine, University of Patras, 26504 Patras, Greece; georg.schinas@gmail.com; 2 3rd Department of Critical Care, EVGENIDIO Hospital, Medical School, National and Kapodistrian University of Athens, 11528 Athens, Greece; gdimop@med.uoa.gr; 3 Department of Internal Medicine and Infectious Diseases, University General Hospital of Patras, 26504 Patras, Greece

**Keywords:** diagnostic stewardship, antimicrobial resistance, resident physicians, healthcare-associated infections, Bayesian reasoning

## Abstract

Antimicrobial resistance (AMR) poses a significant global health challenge, exacerbated by the COVID-19 pandemic. Antimicrobial stewardship programs (ASPs) are crucial in managing this crisis, with diagnostic stewardship (DS) emerging as a key component. DS refers to the appropriate use of diagnostic tests to optimize patient outcomes, improve antimicrobial use, and combat multi-drug-resistant (MDR) organisms. Despite its potential, understanding and application of DS remain ambiguous in multiple respects, which, however, do not directly implicate the implementation of such initiatives. DS is particularly important for resident physicians who are often at the forefront of patient care and can significantly influence future AMR strategies. This review provides a comprehensive overview of DS, discussing its importance, potential challenges, and future directions. It emphasizes the need for resident physicians to understand DS principles and integrate them into their clinical practice from the beginning of their careers. The review also highlights the role of various stakeholders in implementing DS and the importance of continuous education and training. Ultimately, DS is not just a clinical tool but a philosophy of care, essential for a more responsive, humane, and effective healthcare system.

## 1. Introduction

Despite the plethora of advancements in modern medicine, antimicrobial resistance (AMR) continues to be a challenge threatening healthcare worldwide [1]. The CDC’s 2022 special report on AMR in the U.S. revealed that much of the progress achieved during the past decade in combating AMR has been lost, primarily due to the effects of the COVID-19 pandemic, as reflected by the staggering 15% increase in drug-resistant nosocomial (hospital-acquired) infection rates in 2020 compared with the previous year [2]. The report detailed alarming rises in infections attributed to multi-drug-resistant (MDR) bacteria, including a 78% increase in carbapenem-resistant *Acinetobacter*, a 32% rise in MDR *Pseudomonas aeruginosa*, a 14% increase in vancomycin-resistant *Enterococcus* (VRE), and a 13% escalation in methicillin-resistant *Staphylococcus aureus* (MRSA) [3].

The successful management of this global crisis now depends even more heavily upon the prudent prescription and usage of antimicrobials, encapsulated within the efforts of antimicrobial stewardship programs (ASPs) [4]. ASPs are actively seeking to implement their principles of diagnostic testing under the umbrella term of “Diagnostic Stewardship” (DS). DS refers to the appropriate use of diagnostic investigations to ensure optimal patient outcomes, improve the judicious use of antimicrobials, and combat the spread of multi-drug-resistant organisms (MDROs) [5]. Therefore, the concept of DS has emerged as a critical link in this process of vigilance, shaping the core of clinical decisions which govern the administration of these pivotal therapeutic agents.

The principles of DS are of utmost importance when considering the harrowing trends in healthcare-associated infections (HAIs) and the overall inappropriate or excessive use of diagnostic testing [6,7,8]. It mandates meticulous scrutiny of clinical indications before ordering diagnostic tests, considers individual patient characteristics, understands the diagnostic accuracy within different clinical settings, and promotes appropriate and timely diagnostic testing [9]. However, despite the clear potential of DS, ambiguities in comprehensive understanding of the topic and its application persist [10]. Additionally, there exists a continuous demand for updated education, driven not only by the rapidly evolving landscape of diagnostic technologies—ranging from automated task systems to predictive analytics [11]—but also by the inherently complex and nuanced nature of the concepts that it encompasses, i.e., Bayesian reasoning.

For resident physicians, representing the newest links in the chain of antimicrobial stewardship, understanding and mastering the nuances of DS are vital as they are often on the front lines, making initial and critical decisions about patient care. Moreover, being on the cusp of their clinical career, residents are uniquely positioned to embed the principles of diagnostic stewardship into their clinical practice and help shape the future landscape of AMR-tackling efforts. According to a study by Voogt et al., quality improvement educational interventions targeted at the intern level can effectively increase medical residents’ awareness of their organizational roles and empower them to execute small-scale proactive changes in their everyday healthcare practices [12].

The primary aim of this review is to highlight the significance of this concept and provide comprehensive support for early-career physicians, guiding them through the intricate web of decisions they are confronted with and assisting them to be responsible stewards of antimicrobials in their practice right from the start of their medical careers. By providing a robust knowledge base early on in the continuous journey of understanding and implementing diagnostic stewardship strategies, we hope this report can also serve as a potential starting point for quality improvement interventions and policy-making initiatives.

To achieve this objective, a comprehensive search was conducted using databases including PubMed and Scopus. Keywords such as “diagnostic stewardship”, “diagnostic testing”, “antimicrobial resistance”, “antibiotic resistance”, “diagnostic accuracy”, “diagnostic procedures”, “clinical laboratory techniques”, “test utilization”, “predictive value of tests”, “pre-analytical phase”, “analytical phase”, “post-analytical phase”, “evidence-based medicine”, “Bayesian reasoning”, “medical statistics”, and “risk literacy” were used to identify relevant articles published in English. The search was further refined using PubMed’s MeSH (Medical Subject Headings) terms to ensure a comprehensive and focused search. Articles were selected based on their relevance to the core themes of diagnostic stewardship, resident physician education, and future directions for DS. Both primary research articles and authoritative secondary sources such as review articles and guidelines were included to provide a rich context.

The selected articles were analyzed in a narrative manner, with the information being categorized into primary elements of DS, ambiguities, and future directions. By synthesizing these themes, an integrated perspective was developed. The process involved comparing and contrasting different viewpoints, methodologies, and findings to create a coherent narrative that builds a clear and in-depth understanding of the subject. The assistance of a large language model was leveraged to efficiently articulate our concepts and content with optimal clarity and coherence.

## 2. Overview of Diagnostic Stewardship

### 2.1. Understanding the Concept

DS plays a vital role in day-to-day clinical practice, where it guides the ordering, execution, and reporting of diagnostic tests to improve the management of various diseases. By focusing on ordering the right tests for the right patient at the right time, DS provides essential information that streamlines clinical decision making, ensuring that resources are used rationally and that patient care is optimized.

**Figure 1:**
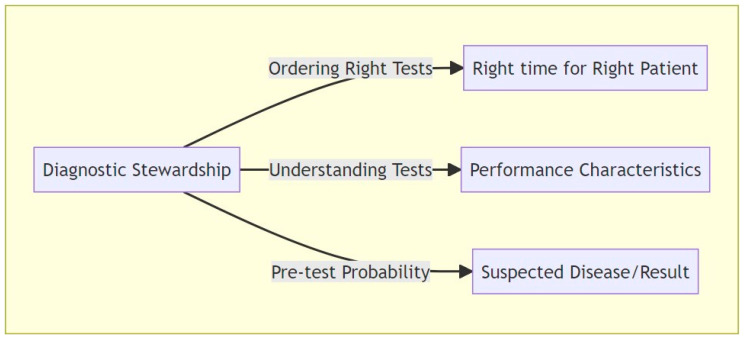
Overview of core principles in diagnostic stewardship (DS). This illustration delineates the fundamental elements of DS, encompassing appropriate test selection, comprehension of test performance metrics, and assessment of pre-test probability.

In the field of infectious diseases, DS involves carefully tailored interventions that promote the efficient use of microbiological diagnostics and enhance infection control. This targeted approach is vital for guiding antibiotic therapy, enabling the prompt initiation and proper termination of antibiotic treatment while also addressing the overuse of anti-infective treatments that often occurs in response to microbiological findings from non-sterile areas [5]. The latter problem is magnified with the increasing use of advanced nucleic acid amplification tests (NAATs), including polymerase chain reaction (PCR) methods, capable of detecting a broad spectrum of viruses and bacteria in panels for respiratory, gastrointestinal, and central nervous system infections, among others [13]. The unselective use of these tests, colloquially termed “shotgun diagnostics”, without considering the pre-test probability of an infection, can rapidly lead to false-positive results and create diagnostic uncertainties as well as cause harm. Conversely, accurate diagnosis is linked to more appropriate antibiotic use [14], leading to fewer adverse events [15] and reduced hospital stays [16]. Therefore, minimizing diagnostic error by implementing DS principles, resulting in fewer false-positive test results and less overdiagnosis, while concurrently identifying true-positive cases enhances overall clinical care [14,17,18]. (Figure 1)

### 2.2. Navigating Ambiguities

While implementing DS, improvement initiatives can be beneficial in many respects, it is important to be aware of potential pitfalls. The main concern is the possibility of missed diagnoses due to improvements in the positive predictive value of testing. However, research shows that, in practice, the actual use of probabilities in diagnostics is often misinterpreted. A survey conducted among 553 U.S. primary care practitioners found a consistent overestimation of the likelihood of diagnosis both before and after tests [19]. Clinicians overestimated the probability of diseases such as pneumonia and urinary tract infection compared to actual evidence-based probabilities. This overestimation even extended to positive and negative test results, where practitioners incorrectly assessed the impact of these test results on the probability of a disease. These findings suggest that such a widespread misinterpretation of probability in diagnostic processes is much more likely to lead to excessive and inappropriate testing, as well as overdiagnosis, rather than the opposite. Nonetheless, it is crucial to remember that DS is designed to support and possibly enhance informed clinical decisions—it should not obstruct personalized treatment strategies. Therefore, tests targeted by stewardship efforts should still be available for special requests or specific condition needs. This ensures that doctors’ ability to provide comprehensive care remains uncompromised.

Additional potential drawbacks of DS initiatives include potential clinician frustration with restrictions or limitations on testing as such guidance could be perceived as a reduction in clinician autonomy. A study stemming from a survey of over 500 clinicians across eight U.S. states presented findings that directly challenge this notion, indicating that the ordering of tests is more about personal decision making and interpretation of clinical situations than a matter of maintaining autonomy. The study identified that aggressive testing practices are more closely linked with clinicians’ cognitive characteristics, namely low numeracy skills, being a medical maximizer—one who pursues a range of health interventions—and a poor understanding of risk in medical practice. Factors such as malpractice fear, low tolerance of risk, and discomfort with uncertainty were also found to be associated with more aggressive testing, though not consistently across all tests [20].

Last but not least, it should be highlighted that the successful execution of effective DS does not solely depend on the perceptions, habits, and background of individual practitioners. Collaboration among various stakeholders, including clinicians, laboratories, administrators, and policymakers is required for successful incorporation of DS principles into regular clinical practice [21]. To this end, the term ‘DS’ itself, originally introduced in 2017, has come under scrutiny by experts in the field. An article composed by the executive committees of two research-specific ESCMID study groups suggested that the term ‘stewardship’ should be reserved for antimicrobials to emphasize the importance of collective impact and shared responsibility in the effort not only to combat resistance but to improve the diagnostic process as a whole [22]. The term’s emphasis on the role of microbiology laboratories and techniques in advancing the application of antibiotics and promoting appropriate diagnostic methods has contributed to this ambivalence. Therefore, it is crucial to acknowledge that the responsibility of improving the diagnostic process for antimicrobial use extends beyond the realm of microbiologists and their laboratories. The diagnostic process is multifaceted and involves clinical evaluation, a wide array of laboratory tests, and various imaging techniques, including X-rays, CT scans, and MRI scans, among others. These elements collectively contribute to the diagnostic process and are not solely tied to microbiology or the laboratory.

## 3. DS Applications

Diagnostic stewardship interventions are categorized into pre-analytical, analytical, and post-analytical domains to reflect the different stages of the diagnostic process and the specific interventions that can be applied at each stage.

### 3.1. Pre-Analytical Domain

**Figure 2:**
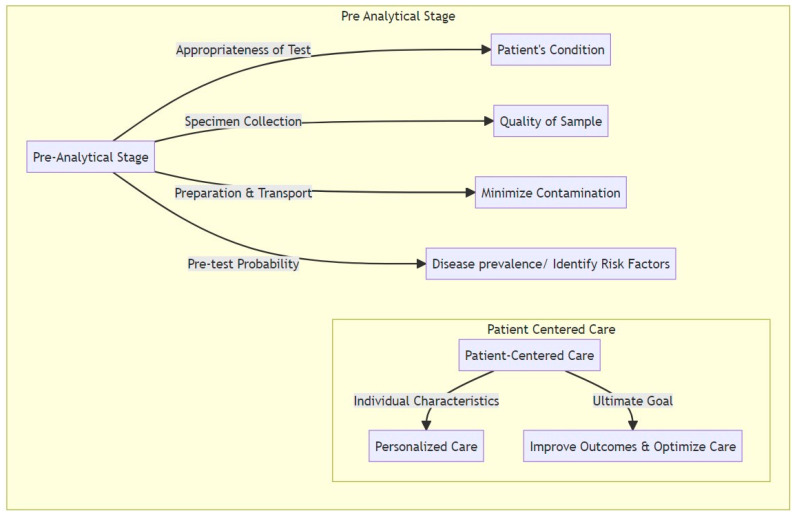
Pre-analytical stage: This stage focuses on the appropriateness of the test, specimen collection, preparation, and understanding pre-test probability. Leveraging patient-centered care is of imminent importance in all domains. The steps of this stage set the groupwork for the subsequent analysis, ensuring accuracy and precision.

Pre-analytical interventions in DS involve several important factors. These include: (1) ordering the appropriate test based on the performance characteristics of the test and the pre-test probability of the suspected disease/result, (2) implementing enhanced or targeted specimen collection techniques, and (3) ensuring optimal preparation and timely transport of the specimen to minimize potential contamination and maximize its quality. (Figure 2)

A key strategy in this phase is optimizing test utilization. This involves ensuring that appropriate tests are ordered, preventing duplicate orders, and enhancing understanding of the intended test use [23]. The integration of a clinical decision support system (CDSS) into an institution’s electronic health records (EHR) can facilitate this process. Additionally, automated laboratory information systems (LIS) can play a significant role in preventing unnecessary or duplicate test orders [21].

However, integrating DS into EHR can present several challenges in terms of increased workload and cognitive overload, with “alert fatigue” causing clinicians to ignore electronic prompts [24]. To address these challenges, it is essential to incorporate diagnostic stewardship into EHR in a manner that reduces redundancy and streamlines routine processes, thereby facilitating accurate and efficient decision making. This may involve careful design of alert systems and ensuring that prompts are relevant and actionable [6]. Furthermore, interventions that change the ease of structural access to tests of interest in these systems, known as “ease of ordering” interventions, may be particularly relevant in this context [25].

Other interventions aimed at specimen processing at the pre-analytical level can include the implementation of stringent criteria for sample rejection by the laboratory for poorly collected materials and specimens. This approach ensures that only high-quality samples are analyzed, thereby increasing the clinical relevance and interpretability of test results. For instance, rejecting well-formed stool specimens for *Clostridioides difficile* PCR testing is a well-known recommendation [26]. Based on the ESCMID guidelines, it is recommended to test only stool samples with a Bristol score of 5 to 7 [27]. However, according to a recent report evaluating diagnostic strategies and laboratory procedures, microbiology laboratories tend to extend testing beyond guideline recommendations due to clinician-initiated requests [28]. Finally, providing education to clinical teams is a fundamental activity that must be undertaken before anything else. This education should not only be physician to physician but must also involve other infectious diseases specialists such as microbiologists. A multi-disciplinary approach is crucial to ensure a comprehensive understanding of novel diagnostic testing methods, new diagnostic algorithms, and changes in current diagnostic guidelines. Regular training sessions and workshops can be conducted to ensure that all staff members are well informed and equipped with the latest knowledge in the field. By fostering continuous interaction and communication between specialists, we can promote a more holistic understanding of antimicrobial resistance and the strategies to combat it.

Aimed at optimizing comprehension of diagnostic stewardship principles, educational efforts at the pre-analytical stage should involve a thorough understanding of the concept of pre-test probability, which is the likelihood of a patient having a disease before a diagnostic test result is known [29]. Although healthcare providers are more statistically literate than the general population, they still struggle to understand, incorporate, and convey Bayesian reasoning [30]. Given its integral part in understanding pre-test probability and predictive values [31], as well as its overall critical role in medicine, there is a compelling need for effective educational interventions to foster comprehension of Bayesian logic among medical professionals. Research suggests that the way statistical knowledge is framed can play a role in facilitating learning [32]. Positive framing of learning objectives in medical statistics education, meaning placing the emphasis on communication-related topics, with statistical content in the background, could potentially improve the teaching and learning of the Bayesian theorem. This approach could help make the Bayesian theorem more accessible and less intimidating, thus encouraging a greater understanding and application of this fundamental concept in both medical practice and patient communication.

In this context, we feel it is imperative to showcase tools that help with the calculation of pre-test probability and foster a better understanding of diagnostic testing principles in general. One such groundbreaking initiative is the “Testing Wisely” website, created by a team led by D.J. Morgan, the researcher who introduced the term diagnostic stewardship. The website’s mission is to fill educational gaps in diagnostic test interpretation by providing interactive tools such as videos, calculators with real testing data, and a “playground” to explore test variables, thereby enhancing the understanding of concepts such as pre-test probability, sensitivity, specificity, Bayesian updating, and predictive values. While the website serves as a valuable educational resource for resident physicians and healthcare professionals seeking to enhance diagnostic quality and decision making, its content is not a substitute for professional medical advice, as indicated in its disclaimer.

### 3.2. Analytical Domain

Analytical interventions involve the actual execution and performance of diagnostic tests. A commonly employed intervention is the use of a reflex testing strategy, where tests are only performed after pre-specified criteria are met. For example, urine cultures are only performed if urinalysis indicates the presence of pyuria or bacteriuria. Studies have shown that the absence of pyuria and bacteriuria in a urine sample has a high negative predictive value for UTI, close to 100% [33]. Therefore, specimens without any white blood cells or bacteria under microscopy are not further processed as these have a low probability of true UTI or significant bacterial growth. Instead, the microscopy result and a rejection comment are reported for the sample in the post-analytic phase. In a recent pre–post multicenter study evaluating the impact of these interventions in urine culturing, it was revealed that DS was independently associated with a 24% reduction in antimicrobial consumption (adjusted odds ratio 0.76, 95% confidence interval (CI) 0.70–0.83, *p* < 0.001) across all healthcare settings. Moreover, DS had no significant effect on patient mortality (adjusted hazard ratio = 0.95, 95% CI 0.89–1.01, *p* = 0.08), and, importantly, no patients with unreported urine culture developed bacteremia from untreated UTI [34]. It is noteworthy that the study automatically cultured specimens from specific patient populations, including those from obstetrics, urology, pediatrics, oncology, or renal transplant wards, as well as urine samples from ureteric, nephrostomy, or suprapubic sources, which were labelled and processed accordingly.

Selective testing and selective reporting are other frequently employed interventions [35]. Selective testing involves not testing antimicrobial susceptibility for a particular pathogen–drug combination on bacteria suspected of being a contaminant. For example, in urine cultures, no further work up is performed if the presence of multiple organisms, i.e., “mixed flora”, is identified. Selective reporting, on the other hand, involves only reporting some part of the results or none of them (suppression of results), for instance, not releasing organism identification if multiple organisms are present in a urine culture. This strategy also involves reporting the resistance findings for the most critical pathogens only for the preferred narrow-spectrum antibiotics, provided they test as susceptible. Evidence indicates that selective reporting methods can be beneficial in decreasing inappropriate and unnecessary antibiotic prescriptions [36]. Some may think that this approach may be viewed as restrictive by clinicians, potentially leading to resistance. However, studies conducted in both inpatient and outpatient settings suggest that prescribers have accepted the implementation of such an intervention for urine culture results [37], with up to 71% of medical interns declaring that selective reporting in fact made their choice of antibiotic easier [38].

“Cascade reporting” (CR) has recently come up as a versatile alternative to selective reporting where antibiotic susceptibility is revealed in a stepwise fashion. The aim of this method is to promote the use of narrower-spectrum antibiotics, when possible, without compromising patient outcomes. In CR, antibiotic susceptibility results for a particular pathogen–drug combination are obtained but suppressed for broader-spectrum agents unless the bug is resistant to narrow-spectrum agents. A study conducted to assess the impact of cascade reporting (CR) on clinical practices revealed that the implementation of CR significantly improves antibiotic de-escalation practices. The percentage of patients whose treatment was de-escalated increased from 48% in the pre-CR period to 71% in the post-CR period. Importantly, this change did not lead to an increase in the length of hospital stay or higher mortality rates [39].

Other methods aimed at the analytical stage involve the introduction of novel molecular diagnostics with optimal performance characteristics in clinical practice to meet the needs of specific patient care pathways. For example, the use of a combination biomarker algorithm that includes procalcitonin (PCT) may provide a potential strategy for evaluating the likelihood of developing bacterial sepsis [40] or guiding the cessation of antibiotic therapy based on PCT kinetics [41].

The utilization of nonculture-based diagnostic testing, especially nucleic acid amplification tests (NAATs), is steadily increasing within daily clinical practice, though primarily in high-income countries [42]. These diagnostic methods can be used for specific or multiplex testing, significantly altering traditional approaches to infectious disease therapy and antimicrobial treatment. Metagenomic next-generation sequencing and applications employing mass spectrometry methods have also been proposed to facilitate broad-range detection and swift diagnosis [43,44]. As with any novel technique, these tests present abundant opportunities and considerable challenges, particularly in standardizing their application within therapeutic algorithms and offering them to clinicians or patients. Each clinical entity has its own unique variations, and each treatment path mandates different considerations in employing these tests [45]. In addition, there is substantial worry about false-positive results and incorrect diagnosis due to the high sensitivity of these tests. This concern is even greater when the tests are used on patients who are unlikely to have the condition before the test is performed [13]. Therefore, adopting a judicious approach and understanding the unique situations of each clinical scenario and how these tests apply in them are of utmost importance.

In the context of bloodstream infections and blood cultures, which represent some of the most commonly ordered but frequently contaminated specimens in the hospital setting [46], rapid diagnostic tools have proved useful in reducing antimicrobial consumption through early de-escalation as compared to conventional follow-up cultures [47]. Moreover, in the case of contaminated blood cultures, mass spectrometry methods can be employed to swiftly identify the causative pathogen and thus reduce the duration of antibiotic therapy [48]. Rapid diagnostic tests have also showcased their value in tackling the issue of MDROs when combined with standard antimicrobial stewardship practices. A study evaluating their combined use in treating patients with bloodstream infections caused by ESBL- and carbapenemase-producing *Escherichia coli* and *Klebsiella pneumoniae* revealed that they significantly reduce the time to optimal and effective antimicrobial therapy compared to conventional microbiological methods with ASP [49].

In settings with a high incidence of MDRO infections or in cases of high-risk individuals, the use of multiplex PCR panels and resistance markers either to identify the causative organism and its resistance pattern or to detect pathogen colonization may be warranted according to some updated algorithms [45]. Nosocomial infections amplify the importance of highly sensitive and specific diagnostic tools. Biofilm formation on implanted medical devices, such as central venous catheters (CVCs), contributes to 50–70% of nosocomial infections [50]. A recent systematic review corroborated that the high prevalence of biofilm-forming microorganisms is linked to an elevated incidence of nosocomial infections among catheterized patients [51]. However, standard blood cultures often fail to identify biofilm-embedded pathogens for multiple reasons, and no standardized protocols are in place for their detection [52,53]. Given that the virulence factors of biofilm-forming species have been well characterized [54], specialized diagnostic approaches—such as molecular techniques—have emerged as promising alternatives for achieving accurate and timely pathogen identification [55,56,57].

Another application of such methods that came to eminence amid the COVID-19 pandemic and proved its clinical significance in preventing unwarranted antimicrobial prescription is the PCR testing for SARS-CoV-2 in identifying pneumonia. Distinguishing viral causes of pneumonia can be challenging, possibly necessitating a variety of diagnostic tests to confidently rule out bacterial pathogens [58]. Similarly, in cases of central nervous system (CNS) infections such as meningitis and encephalitis, the potential applications of highly sensitive molecular rapid diagnostic testing tools and metagenomic sequencing seem invaluable, particularly considering patients might receive antimicrobials before a lumbar puncture or before additional pathogen identification testing is conducted [59,60,61].

**Figure 3:**
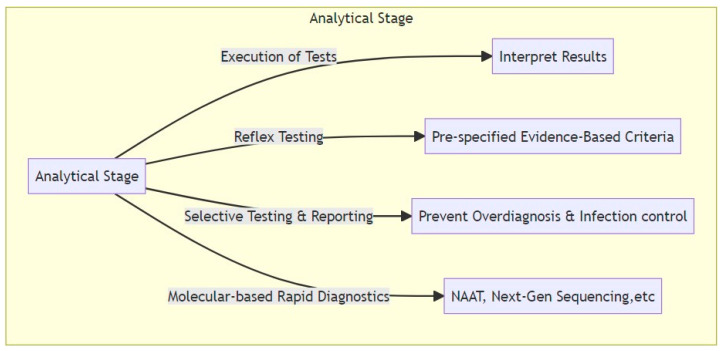
Analytical stage: This stage involves the execution of diagnostic tests, reflex testing, selective reporting, and the use of novel molecular diagnostics. These steps play a pivotal role in translating pre-analytical preparations into tangible results.

Figure 3 delineates the key components and processes involved in the analytical stage.

### 3.3. Post-Analytical Domain

Post-analytical interventions are a vital component of diagnostic as well as antimicrobial stewardship. Ensuring effective and timely communication of results across all levels of the diagnostic process, especially between the laboratory and the clinician, is the core strategy in the post-analytical phase. This involves delivering accurate results in a timely manner to the clinical team. However, studies have shown that the impact of such interventions on care or outcomes may be diminished if results are not paired with appropriate clinical follow up and efficient communication with the microbiology laboratory team [62]. It has become evident that interventions at this level should ideally be integrated into the broader context of an ASP to facilitate and maintain efficient and long-lasting changes to the diagnostic process [63]. A 2017 meta-analysis underscored the importance of this dual and synchronous approach, demonstrating that the use of rapid diagnostics for bloodstream infections significantly decreased mortality risk when paired with an antimicrobial stewardship program but not in its absence [64]. Such an implementation may involve clinical microbiologists providing their expertise and relevant input for clinical management decisions and tailoring interventions to cater to local needs, such as informing testing decisions based on local epidemiology [65].

The use of clinical decision support systems (CDSS) and templated microbiology comments can also aid in the implementation and dissemination of scientific methods to improve sustainability and the adoption of interventions. “Nudges”, as such, have proved their worth in steering the decision making involved in antibiotic prescription while preserving physician autonomy. For example, templated comments on *Candida* spp. in urine cultures as being normal flora, unless high risk, were associated with a significant decrease in antifungal use within the first 72 h of result reporting [66]. Strong and intentional use of assertive phrasing such as “No *MRSA*/No *Pseudomonas* detected” instead of simple annotations such as “physiological flora” in the post-analysis in the case of sputum samples has also been shown to correlate with increased de-escalation tendencies and decreased antibiotic prescriptions [67,68].

Educational efforts are also crucial in post-analytical diagnostic stewardship. Physicians should be educated to interpret test results correctly and evaluate them properly based on evidence-based principles and practices. However, despite the global emphasis in medical schools and institutions on teaching and implementing the principles of evidence-based medicine, there appears to be a deficiency in understanding fundamental concepts of diagnostic evaluation practice such as false positives and positive predictive value within the field. In a study conducted among 4713 OB/GYN residents, only 26% were able to correctly answer a question regarding positive predictive value [69]. This finding is consistent with, and somewhat reflective of, the famous study by Gigerenzer et al. [70], which revealed that a mere 21% of 160 gynecologists could accurately identify the positive predictive value of a screening mammogram. Such statistics underline a significant gap in comprehension that may impact the effective interpretation of diagnostic tests, revealing that basic risk literacy is often lacking. This limitation is problematic given that Bayesian thinking underpins evidence-based medicine [71].

This deficiency highlights a broader issue within medical education, but, promisingly, targeted training can indeed make a profound difference, as a study from Germany exemplified [72]. This study aimed to evaluate the fundamental understanding of medical statistics among medical students and senior educators using a standardized 10-item questionnaire, aptly named the Quick Risk Test, to determine whether inadequacies in statistical literacy could be overcome with training. Involving 169 medical students in their final year and 16 professors of medicine and senior educators, the study found that students initially answered only 50% of the questions correctly compared to the 75% answered correctly by senior educators. Remarkably, a single 90 min training session boosted the percentage of correct answers by students from 50% to 90%, with 82% of participants showing improvement. Focused education can bridge the knowledge gap, providing the foundation for a more precise evaluation of diagnostic testing results and thus a more comprehensive understanding of diagnostic methodology.

**Figure 4:**
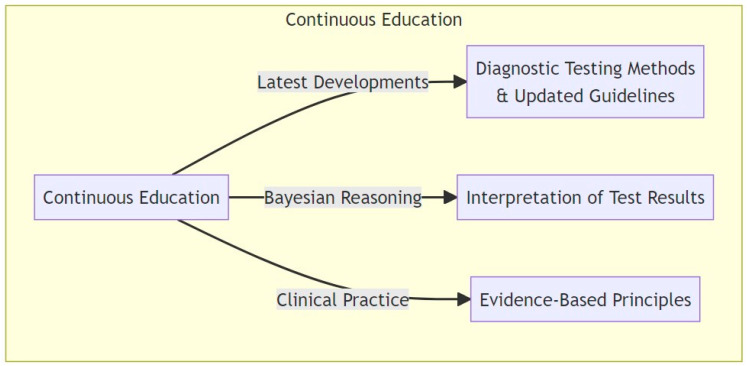
Continuous education: staying updated on diagnostic testing methods and evidence-based practices, and advancing Bayesian reasoning for proper interpretation of test results.

Figure 4 illustrates the components of the continuous education required, emphasizing the need to stay updated on diagnostic testing methods and evidence-based practices while cultivating Bayesian reasoning towards the proper interpretation of test results.

Finally, it is essential to regularly evaluate and adjust the criteria for DS based on emerging potential risks or if the realized benefits do not align with initial expectations. Safety outcomes should always be included in judging the effectiveness of such interventions. This iterative process is fundamental in maintaining the relevance and effectiveness of stewardship programs.

**Figure 5:**
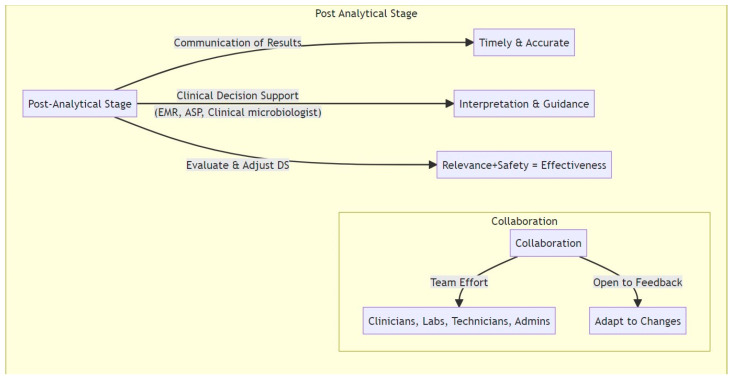
Post-analytical stage: This stage emphasizes effective communication of test results, the use of clinical decision support systems (CDSS), and regular evaluation of DS criteria. Collaboration emphasizes the importance of teamwork among clinicians, laboratories, administrators, and policymakers.

Figure 5 illustrates the post-analytical stage, which ensures that the information is not only derived accurately but is also communicated and utilized effectively.

## 4. Perspectives and Future Directions

Therefore, the future of successful stewardship in diagnostics is likely to be shaped by the integration of dissemination and implementation science (D&I) frameworks. D&I is a relatively new field of research that aims to enhance the quality and effectiveness of health services by facilitating the rapid incorporation of research findings into routine practice [73]. This approach is increasingly recognized as vital in antimicrobial and diagnostic stewardship. It seeks to assess various perspectives and address potential issues that may arise during their implementation [74]. These issues, in the context of a DS intervention, can range from cost considerations and organizational and logistical complexities to the effectiveness of training and the ease of use of testing procedures. Most importantly, however, these frameworks can help investigators to identify pathways to successful applications by addressing barriers to change and evaluating potential harm or adverse outcomes. DS-targeted frameworks can focus on understanding the dynamics of the various elements involved in the diagnostic process and examine their individual impact. For instance, in a laboratory-based diagnostic stewardship intervention, the Practical, Robust, Implementation and Sustainability Model (PRISM) framework can help identify the role and needs of all those involved in the chain of diagnosis, including patients, laboratory, and clinicians, assess all important contextual factors, such as setting, timing, and adverse events, and evaluate the adoption throughout the life of the intervention [75].

In order to provide a practical guide for resident physicians implementing the principles of diagnostic stewardship in their everyday practice, we have created the aid map presented in Table 1. This step-by-step guide is designed to be a comprehensive tool that not only assists resident physicians in implementing the principles of diagnostic stewardship in their everyday practice but also encourages a deeper understanding of their significance in improving patient outcomes and optimizing patient care.

## 5. Conclusions

In conclusion, DS stands as a cornerstone in modern patient care, reflecting a coordinated approach that extends beyond mere testing to embrace an intricate and deliberate process. It plays a pivotal role in guiding therapeutic decisions, optimizing the appropriate use of microbiological diagnostics, and consequently improving patient outcomes. Effective DS mandates a delicate balance of numerous factors. It requires healthcare practitioners to develop a detailed understanding of various factors, including clinical indications, pre-test probabilities, disease spectrums, and the inherent strengths and limitations of diagnostic testing, from the beginning of their career journey.

However, the implementation of effective DS does not solely rest with individual practitioners. Collaboration across various stakeholders—encompassing clinicians, laboratories, administrators, and policymakers—is essential. Coordinated efforts are crucial to overcome existing challenges and to integrate the principles of DS seamlessly into routine clinical practice. This review underscores the need for rigorous training and education in DS starting from the outset of a medical career. It highlights the imperative to build a robust healthcare framework that is sensitive to the intricacies of medical diagnostics. The future of DS resides in the precise alignment of practice with principles, ensuring efficiency and collaboration.

## Figures and Tables

**Table 1:** A roadmap to implementing diagnostic stewardship for resident physicians.

1. Understanding the Concept:
- Diagnostic stewardship (DS) is a coordinated approach to patient care that involves the judicious use of diagnostic tests. It is essential to understand that DS is not just about ordering tests, but about making informed decisions that lead to better patient outcomes;
- DS principles include ordering the right tests for the right patient at the right time, understanding the performance characteristics of different tests, and considering the pre-test probability of the suspected disease/result.
2. Pre-Analytical Stage:
- Before ordering a test, consider its appropriateness for the patient’s condition and the pre-test probability of the suspected disease. This can prevent unnecessary testing and reduce healthcare costs;
- Implement enhanced or targeted specimen collection techniques to ensure the quality of the sample. Poorly collected samples can lead to inaccurate results;
- Ensure optimal preparation and timely transport of the specimen to the laboratory to minimize potential contamination and maximize its quality;
- Pre-test probability is the likelihood of a patient having a disease before a diagnostic test result is known. Understanding this concept is crucial for interpreting test results and making informed clinical decisions.
3. Analytical Stage:
- This involves the actual execution and performance of diagnostic tests. Understanding how tests are performed can help you interpret the results and make informed clinical decisions;
- A reflex testing strategy involves performing certain tests only if pre-specified criteria are met. This can prevent unnecessary testing and ensure that tests are used appropriately;
- Selective testing involves not testing for a particular pathogen–drug combination on bacteria suspected of being a contaminant. Selective reporting involves only reporting some part of the results or none of them (suppression of results). Both strategies can prevent overdiagnosis and unnecessary treatment;
- Novel molecular diagnostics, such as PCR methods and next-generation sequencing, can provide rapid and accurate results. However, they should be used judiciously considering their cost and the need for specialized equipment and expertise.
4. Post-Analytical Stage:
- Effective communication of test results is crucial for patient care. This involves not only delivering accurate results in a timely manner but also explaining the results to the patient and discussing the next steps;
- Clinical decision support systems (CDSS) can aid in the interpretation of test results and guide clinical decision making. Templated microbiology comments can provide standardized interpretations of common test results;
- Regularly evaluate and adjust the criteria for DS based on emerging potential risks or if the realized benefits do not align with initial expectations. This ensures that DS remains relevant and effective in changing healthcare environments.
5. Continuous Education:
- Stay updated on the latest developments in diagnostic testing methods, diagnostic algorithms, and diagnostic guidelines. This ensures that you are providing the best possible care to your patients;
- Bayesian reasoning is a statistical method that involves updating the probability of a hypothesis as more evidence becomes available. It is a fundamental concept in medicine that can aid in the interpretation of test results and clinical decision making;
- Learn how to interpret test results correctly and evaluate them properly based on evidence-based principles and practices. This can prevent misdiagnosis and ensure appropriate treatment.
6. Collaboration:
- DS is a team effort that involves clinicians, laboratories, administrators, and policymakers. Collaboration ensures that DS principles are effectively incorporated into clinical practice and that patient care is optimized;
- Be open to feedback from your colleagues and be ready to adapt to changes in diagnostic practices. This can help you to improve your DS skills and provide better patient care.
7. Patient-Centered Care:
- Always consider the patient’s individual characteristics and needs when making diagnostic decisions. This ensures that the care provided is personalized and effective;
- Remember that the ultimate goal of DS is to improve patient outcomes and optimize patient care. All DS activities should be guided by this goal.

## Data Availability

Not applicable.

## References

[1] Dadgostar P.J.I., Resistance D. (2019). Antimicrobial Resistance: Implications and Costs. Infect. Drug Resist..

[2] Patel J., Sridhar D. (2022). The pandemic legacy of antimicrobial resistance in the USA. Lancet Microbe.

[3] Tanne J.H. (2022). COVID-19: Antimicrobial resistance rose dangerously in US during pandemic, CDC says. BMJ.

[4] Majumder A.A., Rahman S., Cohall D., Bharatha A., Singh K., Haque M., Hilaire M.G.-S. (2020). Antimicrobial Stewardship: Fighting Antimicrobial Resistance and Protecting Global Public Health. Infect. Drug Resist..

[5] Morgan D.J., Malani P., Diekema D.J. (2017). Diagnostic Stewardship-Leveraging the Laboratory to Improve Antimicrobial Use. JAMA.

[6] Curren E.J., Lutgring J.D., Kabbani S., Diekema D.J., Gitterman S., Lautenbach E., Morgan D.J., Rock C., Salerno R.M., McDonald L.C. (2022). Advancing Diagnostic Stewardship for Healthcare-Associated Infections, Antibiotic Resistance, and Sepsis. Clin. Infect. Dis. Off. Publ. Infect. Dis. Soc. Am..

[7] Mrazek C., Simundic A.-M., Salinas M., von Meyer A., Cornes M., Bauçà J.M., Nybo M., Lippi G., Haschke-Becher E., Keppel M.H. (2020). Inappropriate use of laboratory tests: How availability triggers demand—Examples across Europe. Clin. Chim. Acta.

[8] Koch C., Roberts K., Petruccelli C., Morgan D.J. (2018). The Frequency of Unnecessary Testing in Hospitalized Patients. Am. J. Med..

[9] Zakhour J., Haddad S.F., Kerbage A., Wertheim H., Tattevin P., Voss A., Ünal S., Ouedraogo A.S., Kanj S.S. (2023). Diagnostic stewardship in infectious diseases: A continuum of antimicrobial stewardship in the fight against antimicrobial resistance. Int. J. Antimicrob. Agents.

[10] Morjaria S., Chapin K.C. (2020). Who to Test, When, and for What: Why Diagnostic Stewardship in Infectious Diseases Matters. J. Mol. Diagn..

[11] Fabre V., Davis A., Diekema D.J., Granwehr B., Hayden M.K., Lowe C.F., Pfeiffer C.D., Sick-Samuels A.C., Sullivan K.V., Van Schooneveld T.C. (2023). Principles of diagnostic stewardship: A practical guide from the Society for Healthcare Epidemiology of America Diagnostic Stewardship Task Force. Infect. Control. Hosp. Epidemiol..

[12] Voogt J.J., van Rensen E.L.J., van der Schaaf M.F., Noordegraaf M., Schneider M.M.E. (2016). Building bridges: Engaging medical residents in quality improvement and medical leadership. Int. J. Qual. Health Care.

[13] Messacar K., Parker S.K., Todd J.K., Dominguez S.R. (2017). Implementation of Rapid Molecular Infectious Disease Diagnostics: The Role of Diagnostic and Antimicrobial Stewardship. J. Clin. Microbiol..

[14] Morgan D.J., Malani P.N., Diekema D.J. (2023). Diagnostic Stewardship to Prevent Diagnostic Error. JAMA.

[15] Tamma P.D., Avdic E., Li D.X., Dzintars K., Cosgrove S.E. (2017). Association of Adverse Events With Antibiotic Use in Hospitalized Patients. JAMA Intern. Med..

[16] van den Bosch C.M., Hulscher M.E., Akkermans R.P., Wille J., Geerlings S.E., Prins J.M. (2017). Appropriate antibiotic use reduces length of hospital stay. J. Antimicrob. Chemother..

[17] Morgan D.J., Brownlee S., Leppin A.L., Kressin N., Dhruva S.S., Levin L., Landon B.E., Zezza M.A., Schmidt H., Saini V. (2015). Setting a research agenda for medical overuse. BMJ.

[18] McGlynn E.A., McDonald K.M., Cassel C.K. (2015). Measurement Is Essential for Improving Diagnosis and Reducing Diagnostic Error: A Report From the Institute of Medicine. JAMA.

[19] Morgan D.J., Pineles L., Owczarzak J., Magder L., Scherer L., Brown J.P., Pfeiffer C., Terndrup C., Leykum L., Feldstein D. (2021). Accuracy of Practitioner Estimates of Probability of Diagnosis Before and After Testing. JAMA Intern. Med..

[20] Korenstein D., Scherer L.D., Foy A., Pineles L., Lydecker A.D., Owczarzak J., Magder L., Brown J.P., Pfeiffer C.D., Terndrup C. (2022). Clinician Attitudes and Beliefs Associated with More Aggressive Diagnostic Testing. Am. J. Med..

[21] Hueth K.D., Prinzi A.M., Timbrook T.T. (2022). Diagnostic Stewardship as a Team Sport: Interdisciplinary Perspectives on Improved Implementation of Interventions and Effect Measurement. Antibiotics.

[22] Dyar O.J., Moran-Gilad J., Greub G., Pulcini C. (2019). Diagnostic stewardship: Are we using the right term?. Clin. Microbiol. Infect..

[23] Freedman D.B. (2015). Towards Better Test Utilization—Strategies to Improve Physician Ordering and Their Impact on Patient Outcomes. Ejifcc.

[24] Ancker J.S., Edwards A., Nosal S., Hauser D., Mauer E., Kaushal R. (2017). Effects of workload, work complexity, and repeated alerts on alert fatigue in a clinical decision support system. BMC Med. Inform. Decis. Mak..

[25] Barry C., Kaufman S., Feinstein D., Kim N., Gandhi S., Nikolic D., Edmonston T.B., Bierl C. (2020). Optimization of the Order Menu in the Electronic Health Record Facilitates Test Patterns Consistent With Recommendations in the Choosing Wisely Initiative. Am. J. Clin. Pathol..

[26] Baron E.J., Miller J.M., Weinstein M.P., Richter S.S., Gilligan P.H., Thomson R.B., Bourbeau P., Carroll K.C., Kehl S.C., Dunne W.M. (2013). A Guide to Utilization of the Microbiology Laboratory for Diagnosis of Infectious Diseases: 2013 Recommendations by the Infectious Diseases Society of America (IDSA) and the American Society for Microbiology (ASM)a. Clin. Infect. Dis..

[27] Debast S.B., Bauer M.P., Kuijper E.J. (2014). European Society of Clinical Microbiology and Infectious Diseases: Update of the treatment guidance document for Clostridium difficile infection. Clin. Microbiol. Infect. Off. Publ. Eur. Soc. Clin. Microbiol. Infect. Dis..

[28] Bertolino L., Patauner F., Gagliardi M., D’Amico F., Crivaro V., Bernardo M., Scherillo I., Bellitti F., Cusano C., Greco R. (2021). Diagnostic and infection control strategies for Clostridioides difficile infections in a setting of high antimicrobial resistance prevalence. Le Infez. Med..

[29] Sullivan K.V. (2021). Diagnostic Stewardship in Clinical Microbiology, Essential Partner to Antimicrobial Stewardship. Clin. Chem..

[30] Brose S.F., Binder K., Fischer M.R., Reincke M., Braun L.T., Schmidmaier R. (2023). Bayesian versus diagnostic information in physician-patient communication: Effects of direction of statistical information and presentation of visualization. PLoS ONE.

[31] Parikh R., Mathai A., Parikh S., Chandra Sekhar G., Thomas R. (2008). Understanding and using sensitivity, specificity and predictive values. Indian J. Ophthalmol..

[32] Benson S., Schmidt K., Kleine-Borgmann J., Herbstreit S., Schedlowski M., Hollinderbäumer A. (2022). Can positive expectations help to improve the learning of risk literacy? A cluster-randomized study in undergraduate medical students. BMC Med. Educ..

[33] Kayalp D., Dogan K., Ceylan G., Senes M., Yucel D. (2013). Can routine automated urinalysis reduce culture requests?. Clin. Biochem..

[34] Lee A.L.H., Leung E.C.M., Lee M.K.P., Lai R.W.M. (2021). Diagnostic stewardship programme for urine culture: Impact on antimicrobial prescription in a multi-centre cohort. J. Hosp. Infect..

[35] Langford B.J., Leung E., Haj R., McIntyre M., Taggart L.R., Brown K.A., Downing M., Matukas L.M. (2019). Nudging In MicroBiology Laboratory Evaluation (NIMBLE): A scoping review. Infect. Control. Hosp. Epidemiol..

[36] Tebano G., Mouelhi Y., Zanichelli V., Charmillon A., Fougnot S., Lozniewski A., Thilly N., Pulcini C. (2020). Selective reporting of antibiotic susceptibility testing results: A promising antibiotic stewardship tool. Expert Rev. Anti-Infect. Ther..

[37] Le Dref G., Simon M., Bocquier A., Fougnot S., Kivits J., Duda A., Pulcini C., Thilly N. (2023). Selective reporting of antibiotic susceptibility testing results for urine cultures: Feasibility and acceptability by general practitioners and laboratory professionals in France. JAC-Antimicrob. Resist..

[38] Coupat C., Pradier C., Degand N., Hofliger P., Pulcini C. (2013). Selective reporting of antibiotic susceptibility data improves the appropriateness of intended antibiotic prescriptions in urinary tract infections: A case-vignette randomised study. Eur. J. Clin. Microbiol. Infect. Dis..

[39] Johnson L.S., Patel D., King E.A., Maslow J.N. (2016). Impact of microbiology cascade reporting on antibiotic de-escalation in cefazolin-susceptible Gram-negative bacteremia. Eur. J. Clin. Microbiol. Infect. Dis..

[40] Han J.H., Nachamkin I., Coffin S.E., Gerber J.S., Fuchs B., Garrigan C., Han X., Bilker W.B., Wise J., Tolomeo P. (2015). Use of a Combination Biomarker Algorithm To Identify Medical Intensive Care Unit Patients with Suspected Sepsis at Very Low Likelihood of Bacterial Infection. Antimicrob. Agents Chemother..

[41] Iankova I., Thompson-Leduc P., Kirson N.Y., Rice B., Hey J., Krause A., Schonfeld S.A., DeBrase C.R., Bozzette S., Schuetz P. (2018). Efficacy and Safety of Procalcitonin Guidance in Patients With Suspected or Confirmed Sepsis: A Systematic Review and Meta-Analysis. Crit. Care Med..

[42] Apisarnthanarak A., Bin Kim H., Moore L.S.P., Xiao Y., Singh S., Doi Y., Kwa A.L.-H., Ponnampalavanar S.S.L.S., Cao Q., Kim S.-W. (2022). Utility and Applicability of Rapid Diagnostic Testing in Antimicrobial Stewardship in the Asia-Pacific Region: A Delphi Consensus. Clin. Infect. Dis..

[43] Hogan C.A., Yang S., Garner O.B., Green D.A., Gomez C.A., Dien Bard J., Pinsky B.A., Banaei N. (2021). Clinical Impact of Metagenomic Next-Generation Sequencing of Plasma Cell-Free DNA for the Diagnosis of Infectious Diseases: A Multicenter Retrospective Cohort Study. Clin. Infect. Dis..

[44] Beganovic M., Costello M., Wieczorkiewicz S.M. (2017). Effect of Matrix-Assisted Laser Desorption Ionization-Time of Flight Mass Spectrometry (MALDI-TOF MS) Alone versus MALDI-TOF MS Combined with Real-Time Antimicrobial Stewardship Interventions on Time to Optimal Antimicrobial Therapy in Patients with Positive Blood Cultures. J. Clin. Microbiol..

[45] Tiseo G., Arena F., Borre S., Campanile F., Falcone M., Mussini C., Pea F., Sganga G., Stefani S., Venditti M. (2021). Diagnostic stewardship based on patient profiles: Differential approaches in acute versus chronic infectious syndromes. Expert Rev. Anti. Infect. Ther..

[46] Tenderenda A., Łysakowska M., Dargiewicz R., Gawron-Skarbek A. (2022). Blood Culture Contamination: A Single General Hospital Experience of 2-Year Retrospective Study. Int. J. Environ. Res. Public Health.

[47] Rodrigues C., Siciliano R.F., Filho H.C., Charbel C.E., de Carvalho Sarahyba da Silva L., Baiardo Redaelli M., de Paula Rosa Passetti A.P., Franco M.R.G., Rossi F., Zeigler R. (2019). The effect of a rapid molecular blood test on the use of antibiotics for nosocomial sepsis: A randomized clinical trial. J. Intensive Care.

[48] Osthoff M., Gürtler N., Bassetti S., Balestra G., Marsch S., Pargger H., Weisser M., Egli A. (2017). Impact of MALDI-TOF-MS-based identification directly from positive blood cultures on patient management: A controlled clinical trial. Clin. Microbiol. Infect. Off. Publ. Eur. Soc. Clin. Microbiol. Infect. Dis..

[49] Kunz Coyne Ashlan J., Casapao Anthony M., Isache C., Morales J., McCarter Yvette S., Jankowski Christopher A. (2021). Influence of Antimicrobial Stewardship and Molecular Rapid Diagnostic Tests on Antimicrobial Prescribing for Extended-Spectrum Beta-Lactamase- and Carbapenemase-Producing Escherichia coli and Klebsiella pneumoniae in Bloodstream Infection. Microbiol. Spectr..

[50] Asker D., Awad T.S., Raju D., Sanchez H., Lacdao I., Gilbert S., Sivarajah P., Andes D.R., Sheppard D.C., Howell P.L. (2021). Preventing Pseudomonas aeruginosa Biofilms on Indwelling Catheters by Surface-Bound Enzymes. ACS Appl. Bio Mater..

[51] Cangui-Panchi S.P., Ñacato-Toapanta A.L., Enríquez-Martínez L.J., Reyes J., Garzon-Chavez D., Machado A. (2022). Biofilm-forming microorganisms causing hospital-acquired infections from intravenous catheter: A systematic review. Curr. Res. Microb. Sci..

[52] Hall-Stoodley L., Stoodley P., Kathju S., Høiby N., Moser C., William Costerton J., Moter A., Bjarnsholt T. (2012). Towards diagnostic guidelines for biofilm-associated infections. FEMS Immunol. Med. Microbiol..

[53] Silva N.B.S., Marques L.A., Röder D.D.B. (2021). Diagnosis of biofilm infections: Current methods used, challenges and perspectives for the future. J. Appl. Microbiol..

[54] Cangui-Panchi S.P., Ñacato-Toapanta A.L., Enríquez-Martínez L.J., Salinas-Delgado G.A., Reyes J., Garzon-Chavez D., Machado A. (2023). Battle royale: Immune response on biofilms—Host-pathogen interactions. Curr. Res. Immunol..

[55] Wang C., Ye Q., Zhang J., Pang R., Gu Q., Ding Y., Wu Q., Wang J. (2022). Multiplex PCR identification of the major Pseudomonas aeruginosa serogroups using specific novel target genes. LWT.

[56] Usun Jones S., Kee B.P., Chew C.H., Yeo C.C., Abdullah F.H., Othman N., Chua K.H., Puah S.M. (2022). Phenotypic and molecular detection of biofilm formation in clinical methicillin-resistant Staphylococcus aureus isolates from Malaysia. J. Taibah Univ. Sci..

[57] Iorio N.L., Azevedo M.B., Frazao V.H., Barcellos A.G., Barros E.M., Pereira E.M., de Mattos C.S., dos Santos K.R. (2011). Methicillin-resistant Staphylococcus epidermidis carrying biofilm formation genes: Detection of clinical isolates by multiplex PCR. Int. Microbiol..

[58] Virkki R., Juven T., Rikalainen H., Svedström E., Mertsola J., Ruuskanen O. (2002). Differentiation of bacterial and viral pneumonia in children. Thorax.

[59] Moffa M.A., Bremmer D.N., Carr D., Buchanan C., Shively N.R., Elrufay R., Walsh T.L. (2020). Impact of a Multiplex Polymerase Chain Reaction Assay on the Clinical Management of Adults Undergoing a Lumbar Puncture for Suspected Community-Onset Central Nervous System Infections. Antibiotics.

[60] Tansarli G.S., Chapin K.C. (2020). Diagnostic test accuracy of the BioFire^®^ FilmArray^®^ meningitis/encephalitis panel: A systematic review and meta-analysis. Clin. Microbiol. Infect..

[61] Wilson M.R., Sample H.A., Zorn K.C., Arevalo S., Yu G., Neuhaus J., Federman S., Stryke D., Briggs B., Langelier C. (2019). Clinical Metagenomic Sequencing for Diagnosis of Meningitis and Encephalitis. N. Engl. J. Med..

[62] Caulder L., Beardsley J., Palavecino E., Dyke E.V., Johnson J., Ohl C., Luther V., Williamson J. (2018). 1799. Impact of Real-Time Electronic Notifications to Pharmacists of Rapid Diagnostic Blood Culture Results. Open Forum Infect. Dis..

[63] Claeys K.C., Johnson M.D. (2023). Leveraging diagnostic stewardship within antimicrobial stewardship programmes. Drugs Context.

[64] Timbrook T.T., Morton J.B., McConeghy K.W., Caffrey A.R., Mylonakis E., LaPlante K.L. (2017). The Effect of Molecular Rapid Diagnostic Testing on Clinical Outcomes in Bloodstream Infections: A Systematic Review and Meta-analysis. Clin. Infect. Dis. Off. Publ. Infect. Dis. Soc. Am..

[65] Morency-Potvin P., Schwartz David N., Weinstein Robert A. (2016). Antimicrobial Stewardship: How the Microbiology Laboratory Can Right the Ship. Clin. Microbiol. Rev..

[66] Schartz W., Bennett N., Aragon L., Kennedy K., Boyd S.E., Humphrey M., Essmyer C. (2021). Templated Microbiology Comments with Candiduria to Enhance Antimicrobial Stewardship. Open Forum Infect. Dis..

[67] McBride J., Schulz L., Fox B., Dipoto J., Sippel N., Osterby K. (2015). Influence of a “No MRSA, No Pseudomonas ” Comment to a Respiratory Culture in Antibiotic Utilization During the Treatment of Lower Respiratory Tract Infection. Open Forum Infect. Dis..

[68] Musgrove M.A., Kenney R.M., Kendall R.E., Peters M., Tibbetts R., Samuel L., Davis S.L. (2018). Microbiology Comment Nudge Improves Pneumonia Prescribing. Open Forum Infect. Dis..

[69] Anderson B.L., Williams S., Schulkin J. (2013). Statistical literacy of obstetrics-gynecology residents. J. Grad. Med. Educ..

[70] Gigerenzer G., Gaissmaier W., Kurz-Milcke E., Schwartz L.M., Woloshin S. (2007). Helping Doctors and Patients Make Sense of Health Statistics. Psychol. Sci. Public Interest J. Am. Psychol. Soc..

[71] Tiemens B., Wagenvoorde R., Witteman C. (2020). Why Every Clinician Should Know Bayes’ Rule. Health Prof. Educ..

[72] Jenny M.A., Keller N., Gigerenzer G. (2018). Assessing minimal medical statistical literacy using the Quick Risk Test: A prospective observational study in Germany. BMJ Open.

[73] Burnham J.P., Geng E., Venkatram C., Colditz G.A., McKay V.R. (2020). Putting the Dissemination and Implementation in Infectious Diseases. Clin. Infect. Dis..

[74] Feldstein A.C., Glasgow R.E. (2008). A Practical, Robust Implementation and Sustainability Model (PRISM) for Integrating Research Findings into Practice. Jt. Comm. J. Qual. Patient Saf..

[75] McCreight M.S., Rabin B.A., Glasgow R.E., Ayele R.A., Leonard C.A., Gilmartin H.M., Frank J.W., Hess P.L., Burke R.E., Battaglia C.T. (2019). Using the Practical, Robust Implementation and Sustainability Model (PRISM) to qualitatively assess multilevel contextual factors to help plan, implement, evaluate, and disseminate health services programs. Transl. Behav. Med..

